# Recent Insight into SARS-CoV2 Immunopathology and Rationale for Potential Treatment and Preventive Strategies in COVID-19

**DOI:** 10.3390/vaccines8020224

**Published:** 2020-05-14

**Authors:** Sara Lega, Samuele Naviglio, Stefano Volpi, Alberto Tommasini

**Affiliations:** 1 Institute for Maternal and Child Health IRCCS Burlo Garofolo, 34137 Trieste, Italy; sara.lega@burlo.trieste.it (S.L.); alberto.tommasini@burlo.trieste.it (A.T.); 2 Center for Autoinflammatory Diseases and Immunodeficiency, IRCCS Istituto Giannina Gaslini and Università degli Studi di Genova, 16147 Genova, Italy; stefanovolpi@gaslini.org; 3 Department of Medicine, Surgery and Health Sciences, University of Trieste, 34137 Trieste, Italy

**Keywords:** coronavirus, Covid-19, SARS-CoV-2, immune response, T cell repertoire, cytokine storm, antiviral immunity

## Abstract

As the outbreak of the new coronavirus (SARS-CoV-2) infection is spreading globally, great effort is being made to understand the disease pathogenesis and host factors that predispose to disease progression in an attempt to find a window of opportunity for intervention. In addition to the direct cytopathic effect of the virus, the host hyper-inflammatory response has emerged as a key factor in determining disease severity and mortality. Accumulating clinical observations raised hypotheses to explain why some patients develop more severe disease while others only manifest mild or no symptoms. So far, Covid-19 management remains mainly supportive. However, many researches are underway to clarify the role of antiviral and immunomodulating drugs in changing morbidity and mortality in patients who become severely ill. This review summarizes the current state of knowledge on the interaction between SARS-CoV-2 and the host immune system and discusses recent findings on proposed pharmacologic treatments.

## 1. Introduction

Since the beginning of December 2019, the novel SARS-CoV-2 outbreak is spreading globally posing critical challenges for the medical community [1]. So far Covid-19 seems to be more contagious and more deadly than most strains of seasonal influenza. In fact, without containment measures, the basic reproduction number (R_0_) of SARS-CoV-2 has been estimated in the range of 2.2 and 5.7, meaning one case may infect between two and five other persons, while for seasonal influenza this number is reportedly around 1.3 [2,3]. Fatality rates for Covid-19 differ significantly in magnitude across countries. Discrepancies most likely depend, among other factors, on the frequency of asymptomatic or mildly symptomatic patients as well as on the testing strategy adopted in different settings, which may result in a significant proportion of undiagnosed cases. An accurate estimate of the overall infection fatality is still very difficult to calculate. Recent estimates based on aggregate data from China adjusted for demography and under-ascertainment bias suggest that the overall case fatality rate of Covid-19 could be close to 1.38% [4]. However, taking into account the ratio of asymptomatic cases support infection fatality ratios hovering 0.4–0.7% [5]. These figures are lower than that of Severe acute respiratory syndrome (SARS) and Middle East respiratory syndrome (MERS), which had case fatality rates around 10% and 36% respectively [6], yet they seem to exceed those of most severe influenza strains, which have case fatality rates averaging 0.1%. The reasons why some patients progress to severe disease while others only manifest with mild or no symptoms remain to be elucidated. Beside the direct cytopathic effect of the virus, the host hyper-inflammatory response has clearly emerged as a key factor in determining disease severity and mortality.

As clinical and epidemiologic information on SARS-CoV-2 infection have increased, a better knowledge on the burden of severe disease and clinical risk factors associated with disease progression have emerged. Older age, male gender, and pre-existing chronic conditions, such as diabetes, obesity, cardiovascular disease, have been observed to be the most significant risk factors among patients with COVID-19 in China and Europe [7,8,9]. In the United States disproportionately higher rates of hospitalization and death have been reported among African-American and Hispanic groups and may be explained by differences in economic and social conditions of ethnic minorities in specific contexts. Nevertheless, genetic contribution to different clinical outcomes cannot be excluded [10].

Achieving a better understanding of the interaction between SARS-CoV-2 and the host immune system, as well as of the immune pathology driving disease progression, may therefore provide opportunities for treatment interventions and vaccine development.

## 2. What We Know about Immune Response to Coronaviruses

Knowledge about host immune response to SARS-CoV-2 is mostly derived from previous studies of other coronaviruses of the same family (betacoronavirus), namely SARS-CoV and MERS-CoV. Betacoronavirus are positive-sense, single-stranded RNA viruses of zoonotic origin [11]. SARS-CoV-2 genes share approximately 80% homology with SARS-CoV, suggesting that the two viruses belong to the same species. However, it is more likely that SARS-CoV-2 has developed from the bat coronavirus RaTG13, with which it has an even higher degree of homology, close to 96% [12]. 

**Figure 1:**
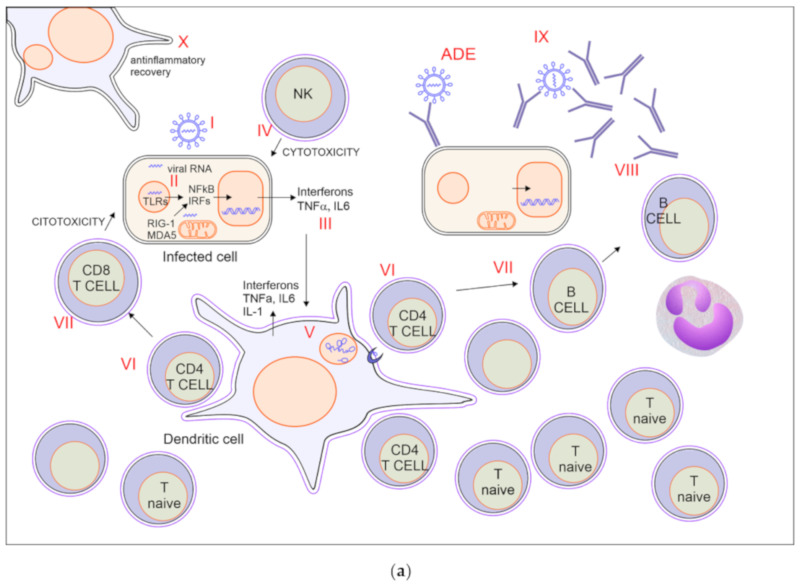

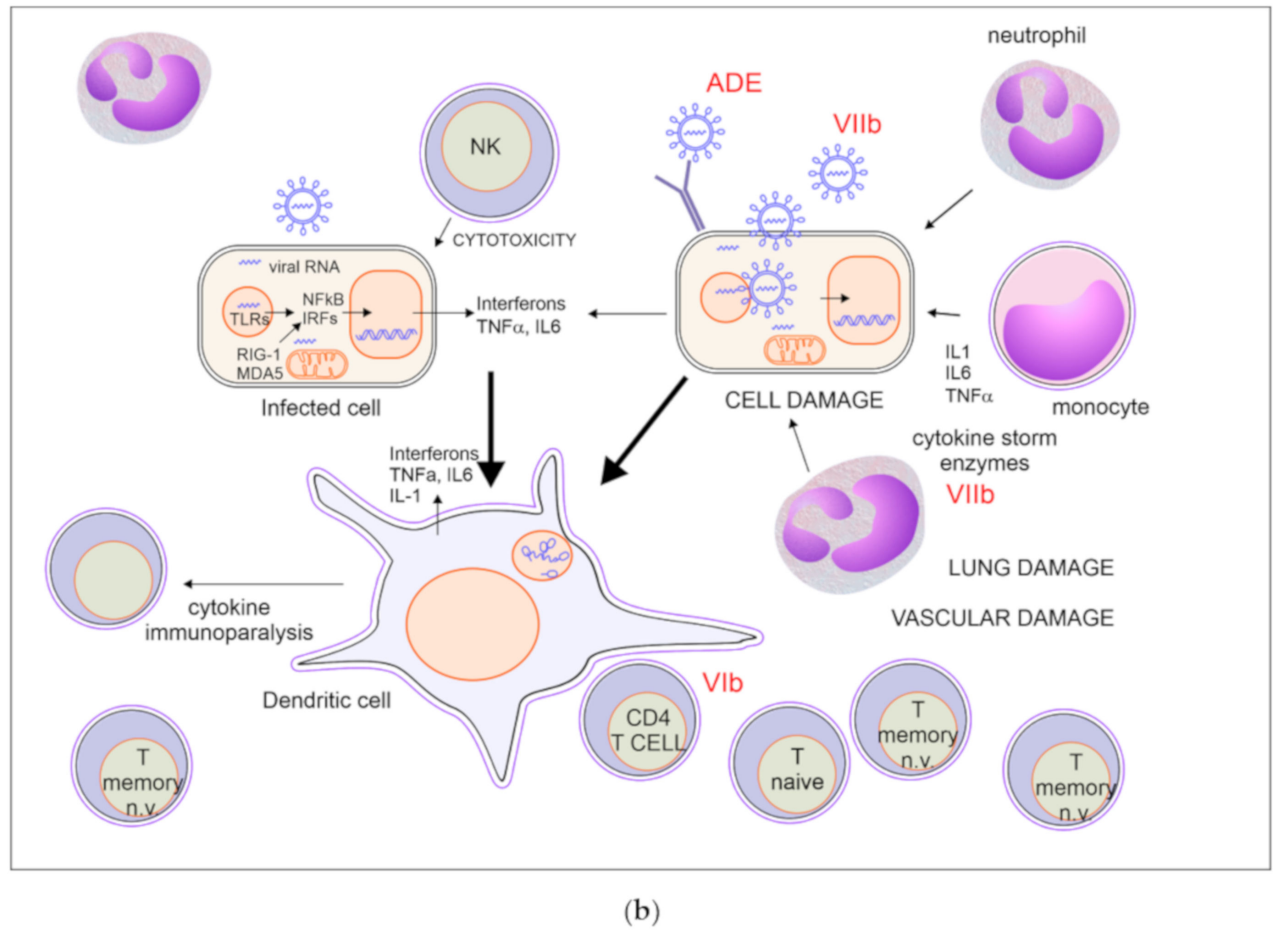
(**a**) Immune response to SARS-CoV-2 with effective recovery. For explanation, see paragraph 2.1. (**b**) Immune response in severe SARS-CoV-2 infection. T memory n.v. = non virus-specific. For explanation, see paragraph 2.3.

Figure 1a illustrates the scenario of a typical immune response to viruses, discussing how it may be relevant to the case of SARS-CoV-2. Even though the proposed sequence may not represent what happens specifically during Covid-19, it describes how distinct immune mechanisms may affect the fate of SARS-CoV-2 infection.

### 2.1. A Possible Scenario of Covid-19 Immune Response with Effective Recovery 

#### 2.1.1. Viral Entry via ACE2 Binding (Figure 1a I)

Both SARS-CoV and SARS-CoV-2 enter human cells exploiting the link with membrane-bound angiotensin converting enzyme II (ACE2) protein, which is widely expressed in many cells across the body, including type II alveolar cells (AT2), upper airways cells, endothelial cells, myocardial cells, proximal tubule cells of the kidney, ileum and esophagus epithelial cells, and bladder urothelial cells [13,14,15,16,17].

Coronavirus spike (S) glycoprotein binds to ACE2 initiating viral entry into host cells. Of note, the affinity of SARS-CoV-2 S protein for ACE2 is higher than that of SARS-CoV [18,19,20]. Whether higher density of ACE2 may facilitate or protect alveolar cells from the infection is still debated [21]. Binding of the S1 subunit of the S protein to ACE2 is followed by the fusion of the viral and cellular membranes mediated by the S2 subunit of S protein and requires S protein priming by cellular proteases. For this purpose, SARS-CoV-2 mainly uses a serine protease, transmembrane serine protease 2 (TMPRSS2), and the endosomal cysteine proteases cathepsin B and L (CatB/L) [16]. Notably, TMPRSS2 is androgen-regulated and this may be related to higher prevalence of both infection and severe disease in males [22,23]. 

After viral entry into cells, expression of membrane ACE2 is downregulated because of endocytosis of the receptor together with the virus. This event results in reduced metabolization and increased levels of angiotensin II in lung tissues and increased stimulation of the Type 1 Angiotensin II Receptor (ATR1), which mediates angiotensin II-induced vascular permeability and severe acute lung injury [24,25]. Notably, injection of SARS-CoV S protein in mice is sufficient to worsen acute lung failure, and this effect is reduced by renin-angiotensin pathway blockage with the AT1R inhibitor losartan [26]. All these observations may explain why SARS-CoV-2 primarily causes pneumonia with vascular injury, differently from influenzavirus, and suggest that higher availability of membrane ACE2 may be protective. Furthermore, activation of ATR1 receptor directly upregulates NF-kB, as well as a disintegrin and metalloprotease 17 (ADAM17) on cell surface; ADAM17 in turn cleaves membrane TNF to soluble TNF and processes membrane IL-6R to the soluble sIL-6R, which allows IL6 responsiveness by many tissues through IL6 trans-signaling, leading to Signal Transducer and Activator of Transcription 3 (STAT3) activation and the amplification of Nuclear Factor kappa B (NF-kB) activation [27]. Once infected, cells are directly affected by SARS-CoV-2 replicative cycle, which is therefore a cytopathic virus causing direct cell death and resulting in increased inflammatory response [28]. Notably, SARS-CoV has been shown to cause caspase-1-mediated cell death (pyroptosis, a highly inflammatory form of cell death) via the activation of the Nod-like receptor family, pyrin domain-containing 3 (NLRP3) mediated by the viral 3a protein which acts as a potassium ion channel (viroporin) resulting in inflammasome activation [29].

Besides alveolar cells, ACE2 is widely expressed also on endothelial cells, macrophages, heart, intestine, and kidney, and this may explain the involvement of these cells in severe cases of Covid-19 [19,30,31]. The ability of SARS-CoV-2 to infect enterocytes has been demonstrated in human small intestinal organoids, and this may explain the relative frequency of gastrointestinal symptoms in COVID-19 [32]. Furthermore, it has been suggested that SARS-CoV2 may persist longer in the digestive system, and this may be a cause of protracted form of disease requiring readmission to hospital in some patients due to gastroenteritis symptoms with persistence of viral RNA in stools after resolution of respiratory symptoms [33].

Recent findings support a primary role of endothelial cell infection and resulting endotheliitis in Covid-19 pathogenesis, which may lead to vasculopathy, coagulopathy, and multiple organ injury [31]. Endothelial injury may be due to direct viral infection of endothelial cells as well as to endothelial activation and apoptosis from inflammatory cytokines, especially tumor necrosis factor alpha (TNFα) [34]. According to recent unpublished data from autopsies, it has been argued that vascular damage with peripheral lung microthrombi may be an early phenomenon directly linked to viral infection rather than to the inflammatory reaction. A significant incidence of disseminated intravascular coagulation and thromboembolism has been also reported. This led to the suggestion to introduce prophylactic heparin in hospitalized patients with Covid-19 [35,36].

Overall, the occurrence of pulmonary vascular (micro)thrombosis and of vascular dysregulation due to angiotensin system abnormalities may account for the observation of severe hypoxemia despite high compliance of the lung, differently to what occurs in most other types of interstitial pneumonia. These findings are likely associated with an increase of dead space ventilation (ventilation of poorly perfused lung), a defective hypoxic pulmonary vasoconstriction with areas not ventilated but perfused (shunt), and may also partly explain the efficacy of proning in patients with respiratory failure [37].

Recent findings demonstrated that SARS-CoV-2 also infect immune cells, causing activation and secretion of inflammatory cytokines [38]. Direct infection of lymphocytes via ACE2 binding had been previously demonstrated for SARS-CoV [39]. Following this observation, it has been said that SARS-CoV could be considered halfway between a common respiratory virus and a lymphotropic virus such as HIV [40]. SARS-CoV-2 seems unable to replicate in lymphocytes, and it is uncertain whether direct lymphocyte infection can contribute to the lymphopenia associated with severe Covid-19. Indeed, lymphopenia may just be due to apoptosis as a part of a multilinear cytopenia induced by hypercytokinemia or by overt hemophagocytic lymphohistiocytosis [7,41]. 

#### 2.1.2. Innate Immune Response (Figure 1a II)

The initial response to coronaviruses infection by the innate immune system plays a pivotal role in determining the outcome of the infection. The sensing of foreign nucleic acids is the first step in the pathway to an effective immune response leading to viral clearance. Eukaryotic cells have several sensors, so-called pattern-recognition receptors (PRRs) that are activated by foreign pathogen-derived material. In the endosomal compartment, Toll-like receptor 3 (TLR3) recognizes double-stranded RNA derived by viral replication, while TLR7, TLR8, and TLR9 recognize respectively single-stranded RNA (TLR7/8) and DNA (TLR9) sequences typical of virus and containing uCpG motifs. In the cytoplasm the two RNA sensors Retinoic Acid-Inducible Gene I (RIG-I) and Melanoma Differentiation-Associated protein 5 (MDA5) recognize double-stranded RNA intermediates produced during viral replication. Activation of cellular sensors elicits the production of type I IFNs and other inflammatory cytokines, which act on the infected cells and on the neighboring cells making them more resistant to the entry of other virus particles and act on resident dendritic cells (DCs) and macrophages to promote the activation and organization of the antiviral response [42]. The redundancy of cell sensors is not surprising given that viruses may develop evasion strategies. For example, SARS-CoV can modify the features of its immunostimulatory RNA, lowering the recognition by MDA5 [43,44]. Moreover, SARS-CoV can also dampen IFN-I production by distinct mechanisms, including degradation of interferon (IFN) pathway components by a papain-like protease [45,46], as well as IFN response by inhibition of STAT1 transport into the nucleus in response to interferon signaling [47]. Similarly, MERS CoV suppresses RIG-I-induced type I and type III IFN production interfering with Tripartite Motif Containing 25 (TRIM25)-mediated RIG-I ubiquitination [48]. 

#### 2.1.3. Production of Interferons (Figure 1a III)

Several evidences have shown that IFN-I production in the very early phase of the infection is a crucial step affecting the course of the disease. In an animal model of MERS-CoV infection, the timing of the IFN-I response directly influences the disease outcome, as early administration of IFN-I protects mice from lethal infection, while delayed treatment does not prevent the development of fatal pneumonia and cytokine storm [49]. Thus, it is logical to assume that a properly functioning pathway from the sensing of viral nucleic acids to IFN production is a prerequisite for an effective antiviral response. The importance of this pathway is also reinforced by the observation that germline defects in proteins involved in the IFN cascade are associated with mendelian susceptibility to severe viral infections [50]. The autocrine effects of IFN include changes of cell membrane composition, with increased content of 25-hydroxycholesterol [51,52] and expression of a set of antiviral genes, such as Interferon Induced Transmembrane Protein 3 (IFITM3) that reduce further cell infection by viral particles [53]. Of note, IFITM3 mutations have been associated with severe influenza cases during H1N1/09 pandemic [54]. The paracrine effect of IFNs involves an antiproliferative activity, mediated by the induction of cyclin-dependent kinases (Cdks) and the activation of several immune cells such as dendritic cells (DCs) that mature, migrate, and increase antigen presentation, natural killer cells (NK) that increase their cytotoxic activity and IFN-γ secretion and T and B lymphocytes inducing Th1 polarization and immunoglobulin-secreting cells differentiation. Notably, interferon response has been found defective in 19% of severely ill subjects, thus supporting a possible pharmacological administration of the cytokine in this subset [55].

#### 2.1.4. Activation of NK-Mediated Killing of Infected Cells (Figure 1a IV)

NK cells have multiple antiviral functions. Upon activation by various stimuli, including IFN-I, they provide rapid killing of virus-infected cells before specific CD8 T cells expansion, and later modulate the adaptive immune response [56]. While NK cells are important in immune defense against DNA-virus, like Epstein Barr Virus or Cytomegalovirus, their relevance is less defined against RNA viruses, and there is no clear data about their role in coronaviruses immune response. NK cell levels in blood from severe SARS-CoV or SARS-CoV-2 patients were lower than normal, but this may not necessarily reflect a shortage of NK in lungs [57,58]. Of note, a trial with third party NK cells to treat Covid-19 is ongoing in China.

#### 2.1.5. Activation of Dendritic Cells, Macrophages, and Neutrophils (Figure 1a V)

Tissue DCs can be activated by cytokines and damage-associated molecular patterns (DAMPs) or can be directly infected by SARS-CoV [59]. In the case of SARS-CoV, it has been shown that the virus fails to actively replicate in DCs, which in turn produce large amounts of inflammatory IFNs [60] and chemokines [59]. Within hours after infection, DCs migrate to the draining lymph nodes, and present viral antigens to virus-specific T cells triggering adaptive immune response [61]. However, it has been hypothesized that SARS-CoV may be able to modulate the secretion of cytokines and chemokines by DCs, among its mechanisms to evade immune response [62]. 

Macrophages and neutrophils are also part of the inflammatory reaction and the balance of their activation may be a crucial point in determining the fate of the infection. Macrophages are thought to be among the major players in the production of inflammatory cytokines associated with Covid-19, including IL-6, TNF-α, and IL-1. Moreover, it has been hypothesized that infected macrophages expressing ACE2 could migrate to blood and spleen contributing to the spread of the infection [63]. Liao et al., analyzing cells from bronchoalveolar lavage of patients with Covid-19, showed that severe disease course was associated with a prevalence of monocyte-derived macrophages that overwhelmed tissue resident macrophages and produced large amounts of cytokines involved in the inflammatory storm typical of the disease. Conversely, in milder cases, tissue resident macrophages contributed to the expansion of clonal CD8 T cells [64]. Interestingly, in a mouse model of SARS-CoV infection, removal of inflammatory macrophages protected the animals from lethal infection, without affecting viral load [65], whereas removal of neutrophils led to no improvement in viability. On the contrary, removal of neutrophils in a mouse model of severe influenza hindered viral clearance. In this model, ablation of IL-6 accounted for a similar effect, since the cytokine has antiapoptotic effects on neutrophils [66]. Although this model may not apply to coronavirus infection, it raises the possibility that an excessive inhibition of IL-6 could delay the clearance of the virus also in SARS. 

#### 2.1.6. Recruitment and Activation of Virus Specific CD4 T Cells (Figure 1a VI)

At this point, the large majority of infected subjects can recover from the infection owing to a balanced immune response. In Figure 1a, in the steps from lymphocyte activation (VI) to production of anti-inflammatory mediators (X), we try hypothesizing a possible process leading to resolution of the infection, based on analogies to what we learnt from other viral infections. 

Recruitment of antiviral T cells is thought to occur mainly in draining peribronchial lymph nodes, where DCs bring viral antigens. The process of recruitment and expansion of lymphocytes is likely to start some days after virus infection and to take about a week in normal conditions. A crucial aspect to contrast the spreading of the infection is the availability of a large T cell repertoire, with several distinct precursors that can be clonally expanded. Indeed, rapid expansion of sufficient T cells to contrast the virus spreading is particularly important, as during the kinetics of the infection the coronaviruses tend to dampen T-cell response by several distinct mechanisms, including inhibition of DCs functions [67]. Several studies suggested that the wider T cell repertoire in children and young adults account for a prompt and effective response to novel viruses, for which there is no acquired specific memory to be recalled [68]. 

Of note, in SARS-CoV and MERS-CoV infection, airway CD4 T cells directed against a shared epitope between the two viruses mediated a protective memory immunity, which was dependent on the production of IFN-γ [69]. The development of a shared vaccine based on common conserved epitopes has been proposed as a strategy to fight emerging respiratory coronaviruses. 

#### 2.1.7. Activation of CD8 Mediated Cytotoxicity and B Cell (Figure 1a VII)

Virus-specific CD4 T cells sustain the activation of cytotoxic CD8 T cells, which together with NK have particular importance in the killing of infected cells. Moreover, virus-specific CD4 T cells promote the production of antiviral antibodies by B cells.

#### 2.1.8. Production of Antibodies (Figure 1a VIII)

In most cases, antiviral antibodies are considered able to block further spreading of the virus to other organs and to provide prompt defense against new challenges with the same virus. Moreover, administration of hyperimmune plasma from recovered patients has been proposed to treat patients with severe Covid-19 [70]. However, the development of protective antibodies is not strictly required to overcome the disease, since two subjects with congenital agammaglobulinemia spontaneously recovered from Covid-19 pneumonia [71]. In contrast, subjects with common variable immunodeficiency, who have defective but not absent B-cell function, tend to develop more severe disease, requiring multiple drug treatment [72]. This may suggest that B cells may also be implied in severe Covid-19 pathogenesis. Non-protective antiviral antibodies may also be produced, which could even enhance viral entry in cells expressing the Fc receptor for immunoglobulins [73]. This phenomenon, named ADE (antibody dependent enhancement of viral entry) has been well described for other viruses and represents matter of concern for the development of vaccines. For example, engineering of SARS-CoV antigens has been proposed to select peptides inducing blocking antibodies but not ADE [74]. So far, though, ADE has not been demonstrated for SARS-CoV-2. Interestingly, ADE has been demonstrated to occur both in subjects previously exposed to the same virus and in subjects exposed to viruses from the same family. For example, subjects who recovered from a first infection with Dengue virus may get infected again with worse course because of the presence of enhancing antibodies [75]. Similarly, subjects who recovered from Dengue may undergo a severe course from Zika virus infection and vice versa [76]. The occurrence of ADE has been hypothesized also in subjects with severe course of Covid-19 in China, in areas that were previously hit by the epidemics of SARS-CoV [77].

Apart from antiviral response, other kinds of antibodies may be involved in the modulation of Covid-19 immunopathology. Liu et al. showed in an animal model of SARS-CoV infection that the presence of anti-spike IgG actually promoted proinflammatory monocyte/macrophage recruitment and lung injury. Moreover, sera from SARS-deceased patients enhanced monocyte chemoattractant protein 1 (MCP1) and IL-8 production by human monocyte-derived macrophages, which was reduced by blockade of FcγR [78]. Similarly, mounting evidence suggests that vasculitis-related antibodies, e.g., antiphospholipid antibodies, may complicate and worsen the vascular inflammation [79,80]. Interestingly, several clinical observations both with SARS-CoV and SARS-CoV-2 suggest that natural anti-blood group antibodies may also play a role in the infection, as O blood type could represent a protective factor as compared to A blood type [81,82], and that anti-A antibodies could block the interaction of S protein with ACE2 [83]. 

#### 2.1.9. Block of Viral Spreading (Figure 1a IX)

Altogether, NK cell and CD8 T cell-mediated killing of infected cells, clearance of virus inside macrophages, prevention of viral spreading by antibodies, may contribute to the containment of the infection.

#### 2.1.10. Anti-Inflammatory Recovery (Figure 1a X)

The recovery of viral infections is associated with switch-off mechanisms, which include production of anti-inflammatory cytokines and apoptosis of infiltrating immune cells [84].

### 2.2. A Possible Scenario of Immune Response in Severe Covid-19

Hereafter, for explanatory purposes, we will consider the scenario in which, due to old age or to immunodeficiency, the virus specific T cell repertoire is too small to rapidly overcome virus proliferation (Figure 1b). For the sake of simplicity, we consider that the first steps of innate immune response do not change.

#### 2.2.1. Insufficient Recruitment of Virus-Specific T Cells (Figure 1b VI)

For reasons that are not fully understood, some people fail to carry out the immune response properly. Since old age is the main risk factor for a severe course of the infection, we hypothesized that the reduced T cell repertoire associated with immune senescence accounts for a delayed adaptive immune response in elderly subjects, similarly to what has been reported for pandemic influenza or other viruses. Indeed, elderly subjects may fail recruiting enough virus-reactive lymphocytes to contrast infections from viruses for which they do not have immunological memory. This may be only one of the reasons why some patients develop severe courses of disease. Other factors affecting the course of the infection may include the production of non-neutralizing antibodies enhancing viral spreading and entry into cells (by ADE mechanism), or the worsening of vascular damage by autoantibodies. Impaired innate immunity, either due to ageing, virus-induced mechanisms of immune evasion, and possibly individual genetic differences, also contributes to inadequate adaptive immune response. It has been demonstrated, in fact, that blocking IFN-I signaling in MERS-CoV infection impairs virus-specific T-cell responses, leading to increased inflammation and altered cytokine response [49]. Moreover, a slow immune reaction may not be able to eradicate the infection in subjects affected with various comorbidities that can reduce the functional reserve necessary to cope with a prolonged multiorgan disease.

#### 2.2.2. Virus and Inflammation Induced Tissue Damage (Figure 1b VII)

Tissue damage in Covid-19 is associated both with direct cytopathic action of the virus on infected cells [28,85] and with excessive activation of immune reaction with the release of enzymes and cytokines. Interestingly, depletion of lymphocytes and increase of neutrophils in peripheral blood are changes typically associated with worsening of disease course. Although direct infection may account for increased lymphocyte apoptosis, it seems more likely that depletion of lymphocytes depends on the production of inflammatory cytokines, similarly to what has been described for other viral illnesses, even if lymphopenia may also partly be due to accumulation of lymphocytes in diseased lungs [86,87,88,89]. For example, a direct correlation between serum IFN-α and lymphocyte depletion was described in pigs with swine flu [90] and confirmed in experimental models of viral infection in mice lacking type I interferon receptor IFNAR [91]. Thus, the shortage of virus-specific lymphocytes may even be worsened by cytokine mediated immune paralysis. Notably, lymphocytes from patients with severe Covid-19 often present an exhausted phenotype [92] and this may reflect a functional impairment. Assessment of the immune response in a group of patients with severe Covid-19 with respiratory failure highlighted features supportive of either lymphocyte depletion with low Human Leukocyte Antigen -DR (HLA-DR) expression on monocytes, or macrophage activation syndrome. Subjects with macrophage activation syndrome had higher serum C reactive protein (CRP), ferritin, and IL-1β levels and lower white blood cell count than those with lymphocyte depletion. Conversely, subjects with lymphocyte depletion had higher levels of IL-6 and a trend for higher TNF-α. In these patients anti-IL6 treatment with tocilizumab resulted in increased circulating lymphocytes, highlighting the role of cytokine storm in the immunopathogenesis of the condition [93]. However, since in at least a subset of severe cases has been shown an impaired type I interferon activation, it might be possible that lymphopenia persistence in this setting might be type I interferon independent. 

On the contrary, increase of neutrophils is associated with tissue damage and cytokine storm. Recent data based on autopsy findings support the possibility that the release of neutrophil extracellular traps (NETs) by neutrophils may contribute to the phase of amplification of organ damage and mortality in Covid-19, leading to hypothesize a therapeutic role for medication inhibiting this process, such as DNase, neutrophil elastase inhibitors, IL-1 targeted therapies and colchicine [94].

**Figure 2:**
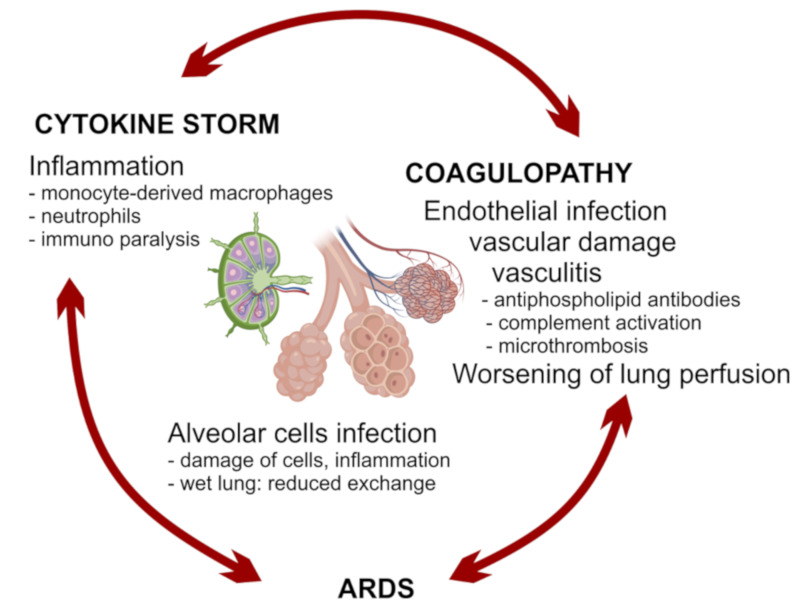
A hypothetical vicious circle between distinct pathogenic mechanisms associated with disease worsening.

The vicious circle between vascular damage, lung damage, inflammation, and immune paralysis can rapidly be responsible for fatalities, especially when patients cannot endure the challenge of the infection on vital functions, because of comorbidities (Figure 2). Acute respiratory distress syndrome (ARDS) can result from alveolar cell damage and lung inflammation and can be worsened by ACE2 depletion, lung vasculopathy with micro-thrombosis [95]. The diseased respiratory environment is also exposed to the risk from secondary bacterial or fungal infections, which can precipitate a fatal course of the infection [7]. Heart or kidney failure can also occur in severe cases [96]. A crucial aspect of the disease is coagulopathy that can accelerate the progression toward multiorgan failure [97]. Vasculopathy and coagulopathy are probably related to the infection of endothelial cells by SARS-CoV-2 [31,98], but it could be worsened by the development of vasculitis, with the production of autoantibodies, such as antiphospholipid, and activation of complement [79,80,99].

## 3. Immunity to SARS-CoV-2 and Factors Determining Disease Progression

There are probably distinct factors affecting the disease progression in Covid-19. The stronger risk factor lies in aging, with a continuous gradient of increasing fatalities more evident in subjects above 50 years of age. Of course, higher incidence of comorbidities also plays a crucial role in these subjects, and most healthy elderly people will survive the infection. However, case fatalities are described also in previously healthy young people and there is great uncertainty as concern genetic and environmental factors that account for these severe cases. Here we discuss some of these factors in greater detail.

### 3.1. The Age Gradient

The risk of severe disease and death in Covid-19 increase directly with age. This increase is particularly significant in the elderly compared with children and young adults. As recent estimates have shown, the fatality rate is considered relatively low, around 0.31% in the population below the age of 60, while it starkly increases to 6.4% among people ≥60 years and up to 13.4% ≥80 years [4]. Based on current knowledge, children infected with SARS-CoV-2 are less likely to be symptomatic or develop severe symptoms [21,100]. Reasons for this difference remain hypothetical and may be related to age-dependent immune factors as well as dynamics of viral exposure [21]. 

#### 3.1.1. ACE2 Density and Distribution Changes with Age

Higher expression of ACE2 receptors may partly explain why young people seem to be less susceptible to severe infections from SARS-CoV-2 [101]. Besides age, several other factors also seem to modulate ACE2 expression, including diet, sexual hormones, drugs, glucose metabolism. Smoking upregulates the expression of ACE2; this may account for a lower than expected prevalence of smokers in diseases cohorts, even though the overall role of smoking as a risk factor is still unclear [102]. Studies in animal models have suggested that ACE inhibitors and angiotensin-receptor blockers (ARBs) upregulate ACE2 expression [25]. Those observations were used to speculate on a possible increased susceptibility to SARS-CoV-2 infection in patients under those treatments, or vice versa, if they could have a therapeutic role. However, three large studies (a database study [103], a case-control study [104] and an electronic medical record analysis [105]) including a total of more than 21,000 infected patients failed to identify any correlation between ACE inhibitor or ARBs treatment and infection risk or disease severity. Scientific societies advice not to discontinue such therapies on the base of Covid-19 fear or actual disease.

#### 3.1.2. T Cell Repertoire and Aging

With aging, several important changes in the innate and adaptive immune responses occur that can explain why older people are more prone to develop severe disease during viral infections. This is particularly true for newly arising infectious organisms, for which there is no protection from pre-existing cross-reactive antibodies generated after previous exposures to related viruses.

Age-related changes in bone marrow result in decreased lymphopoiesis and increased output of functionally compromised myeloid cells. In thymus, tissue involution results in loss of naive T cells and contraction in T-cells repertoire diversity [106]. Studies in animal models showed a decline in reactivity to viral epitopes with a low naive precursor frequency in aged mice [107]. Gene expression analysis in critically ill patients with influenza suggest an impaired development of adaptive immunity leading to an unremitting cycle of viral replication and innate cytokine-chemokine release [108]. Coherently, a relatively better performance of children and elderly in contrasting the 2009 H1N1 influenza pandemic has been explained respectively by a wider lymphocyte repertoire in children and immune memory for past cross-reacting viral strains in old people.

Interestingly, the repertoire of naive T cells persists with aging in a homeostatic balance with memory T cells and can be reduced by the involvement of adaptive immune system in recurrent infections [109]. Whether immunosenescence associated with aging, and specifically T cell repertoire reduction, plays a significant role in the susceptibility of old people to SARS is still uncertain [21].

#### 3.1.3. Age-Related Changes in Antibody Production

Deterioration of immune response in elderly represents a well-known challenge to vaccine developers as immunosenescence also involves antibodies production. The contraction of B-cell repertoire with aging is less impressive than that of T cells [110]. Moreover, while T cells reactivity is restricted to specific peptides, antibodies frequently display some degree of cross-reactivity between distinct antigens. Consequently, old people can boost the production of antibodies directed against antigenically related viruses when exposed to an infection, maintaining a wider immunological memory. This mechanism has a role in the antibody response to influenza virus, where cross-reactive immune responses have been well described [111]. Also, as recent findings have shown, binding and blocking antibodies to common coronaviruses are higher in old adults than in the younger [112].

The ability of making antibodies, also exploiting cross-reactive memory responses, could be a double-edged sword. Cross-reactive antibodies are not always beneficial, and in some cases they can do harm by enhancing inflammatory immune responses and viral entry into cells [113,114]. However, even antibodies mediating viral infection can be protective in certain circumstances [115]. Even if the production of antibodies undergoes significant changes with ageing, there is not any proof that these changes are related to the higher severity of Covid-19 in the elderly.

#### 3.1.4. Innate Immune System and Ageing

The innate immune system also becomes dysregulated and is characterized by heightened levels of basal inflammation, because of increased baseline cytokine production, and failure of activation of innate immune mechanisms in response to pathogens or vaccines [116]. Cells from older donors infected in vitro by influenza and West Nile virus show dysregulated TLR-signaling in monocytes, macrophages, and blood DCs, impaired IFN production, and diminished induction of late-phase antiviral responses [117]. As mentioned before, IFN-I response has been shown to be key in determining outcomes in MERS CoV infected mice [49]. Thus, it could be hypothesized that in the elderly dysregulation of IFN pathways could contribute to lethality from SARS-CoV-2 infection by determining the inability to control viral replication at the beginning of the infection, and later facilitating the occurrence of a deleterious exaggerated inflammatory response.

### 3.2. Why Do Some Young People Become Critically Ill?

#### 3.2.1. Viral Load

In experimental conditions, the inoculum dose is a crucial factor influencing the outcome of the infection from many viruses. High inoculum dose of viruses with direct cytopathic effect can lead to severe damage and death before adaptive immune response develops, especially with fast-replicating viruses. In influenza virus infection, cytotoxic CD8 cells are the main player in killing virus-infected cells. However, with high viral inoculum dose cytotoxic CD8 cells can be killed by DCs, depending on the cytokine environment [118]. In an in vitro model to examine the effects of influenza infection on DC function, T-cell proliferation occurred at low multiplicities of infection, while at higher doses interaction between DC and T cells was defective, partly because of hyperproduction of Transforming Growth Factor beta 1 (TGFβ1) by DCs [119]. Conversely, extremely low doses do not infect DCs and can resolve without engaging the adaptive immunity [84].

#### 3.2.2. Variants in Innate Immunity Genes

Point mutations in genes encoding for proteins involved in DNA sensing and IFN response, as mentioned before, have been found to confer susceptibility to a narrow range of virus infections or even one particular virus. Such an example is that of mutations in genes that participate in the TLR3 signaling pathway which predisposes to severe presentation of herpes simplex virus (HSV), including HSV encephalitis [50]. Whether patients who develop lethal Covid-19 infections harbor genetic variants predisposing to disease progression is unknown. However, this hypothesis is being investigated in genetic studies on young patients developing severe disease.

#### 3.2.3. Immunodeficiencies Affecting T Cell Repertoire

It is well-known that subjects with combined immunodeficiencies (CID) are susceptible to severe illnesses from various viruses [50]. Accordingly, primary immunodeficiencies might explain a proportion of the rare Covid-19 pediatric cases requiring intensive care. However, only a few CIDs can remain undiagnosed for a long time, and thus it is unlikely that subjects with these disorders can encounter SARS-CoV-2 and develop a severe disease course. Indeed, the only records of CIDs detected after severe viral illness concern subjects with undiagnosed idiopathic CD4 T-cell lymphopenia who developed severe varicella [120] or subjects with Cartilage Hair Hypoplasia [121]. Covid-19 could also have a worse course in subjects with not-virologically suppressed HIV, while no fatality has been reported in a small series of young patients on highly active antiretroviral therapy [122].

#### 3.2.4. Immunodeficiencies Affecting Cytotoxic Functions

Severe cases of Covid-19 have been associated with the development of secondary hemophagocytic lymphohistiocytosis (sHLH). Inborn errors of immunity affecting NK and CD8 T-mediated cytotoxicity have been associated with unexpected fatal infection from various viruses, presenting with the clinical picture of primary HLH [123]. Thus, it is possible that some patients develop severe illness because of defective cytotoxic functions. Of note, HLH-related mutations are relatively rare in the population and therefore they are not likely to explain a significant proportion of severe cases. Patients with rheumatic conditions such as systemic juvenile idiopathic arthritis and systemic lupus erythematosus who develop sHLH-like inflammatory complications (named macrophage activation syndrome) are more likely to carry heterozygous variants in genes mediating the release of cytotoxic granules from NK cells and CD8 T cells [124], however their pathogenic role is unclear. To date, no case fatality from SARS-Cov-2 has been attributed to a monogenic primary immunodeficiency.

#### 3.2.5. HLA Haplotypes Correlations

Bioinformatic tools have already shown the different affinities of human leukocyte antigen (HLA) molecules haplotypes to SARS-CoV-2 epitopes [125]. Those differences determine the quality of the immune response, therefore might have a role in vaccine design (i.e., to choose the most appropriate epitope in term of immune response to vaccine) and to provide information to identify subjects with increased risk of severe disease and guide social interventions. For example, HLA-B*46:01 haplotype has been predicted to be a risk factor for a severe disease, as already shown by genetic population analysis in patients affected by severe SARS-CoV [126]. On the opposite side, B*15:03 allele is predicted to have high affinity for epitopes shared by different coronavirus, thus representing a possible protective factor for disease severity through cross-response. To identify high risk populations is of key relevance to implement stratified social distancing rules and disease prevention (i.e., vaccine administration) priorities. To date, there are no population studies on Covid-19.

## 4. Why Bats Don’t Get Sick and What They Can Tell Us

Even though there is no definitive explanation of how SARS-Cov-2 has arisen, hypotheses point toward a spillover from bats, likely mediated by some other animal species as intermediate host [127]. Bats are known to be asymptomatic reservoirs for many different viruses that cause serious disease in humans and non-human primates such as SARS and MERS coronaviruses, Nipah and Hendra paramyxoviruses, and Marburg and Ebola filoviruses [128,129,130,131].

Understanding how can bats coexist with so many viruses with no overt disease could provide guidance for the identification of treatment targets in humans. One hypothesis is that the bat immune system coevolved with viruses mitigating cellular pathways activated by viral infection to reach a state of reciprocal tolerance. Bats may be capable of limiting the infection-induced immunopathology even in the most highly infected tissues as a result of these unique adaptations at the cost of infection persistence and prolonged viral shedding [132], which is probably a prerequisite for viral spillover to other species. Bat cells are capable of containing virus propagation by inducing an effective IFN production in response to viral RNA, while limiting the expression of virus-induced inflammatory cytokines. As gene-expression analyses have revealed, bat cells have varied and tightly regulated expression patterns of different IFN stimulated genes (ISGs). Compared to human cells, bat cells have higher baseline ISGs expression levels and upon IFN stimulus show a higher peak but also a more rapid decline in ISGs expression levels [133]. These unique features are only one of several mechanisms that bats have developed to prevent excessive inflammation. Several species of bats have reduced production of TNF-α [134] and adaptations in NK cell receptors signaling pathways that are associated with inhibitory responses [132].

Interestingly, bat immune cells also show significantly dampened activation of the NLRP3 inflammasome in response to RNA viruses [135]. NLRP3 is an important sensor for cellular stresses as well as viral and bacterial infections. NLRP3 regulates the secretion of proinflammatory cytokines interleukin 1 beta (IL-1β), a key cytokine in the development of inflammatory syndromes such as macrophage activation syndrome. SARS-CoV and MERS-CoV have been shown to induce NLRP3 inflammasome in mouse and human cells with multiple mechanisms [29] and this activation might play a key role in the infection-induced hyperinflammation and immunopathology [136,137,138,139]. 

## 5. Critical Mechanisms of Potential Relevance to Determine the Course of Infection and Possible Targets of Therapies

A good balance between protective immunity and inflammation is crucial to overcome the infection. Prompt recruitment and expansion of virus-specific lymphocytes can lead to early viral clearance, preventing the development of significant organ damage. Conversely, slower activation of specific immunity may result in undisturbed viral replication, with widespread cell damage and secondary inflammatory amplification. Notably, SARS-CoV2 is a fast-replicating virus, reaching viral load peak in the upper airways within 5–6 days from symptoms onset, thus significantly earlier than SARS-CoV, which peaked at about 10 days, therefore a prompt and adequate immune response is crucial to avoid infection progression [140,141].

All the components of immunity may act as friend or foe: IFNs are necessary in the first phase of the infection but may become harmful afterwards; tissue macrophages and DCs are important players in creating the correct local environment to defeat the virus, but they can be replaced by monocyte-derived macrophages with a stronger inflammatory activity and with the risk of bringing the virus to other organs; neutrophils are needed for viral clearance in animal models of infection with influenza virus, but are primary players of tissue damage in Covid-19; antibodies may be protective or harmful by enhancing viral entry or by targeting vascular structures. Healthy people may cope with an imbalance in these mechanisms until proper anti-inflammatory, anticoagulant, and supportive treatments are given. On the contrary, people with older age and comorbidities may pay the higher fatality rate because of the establishment of vicious circles between inflammatory, respiratory and vascular processes. A possible list of critical mechanisms affecting the fate of Covid-19 is proposed in Table 1.

## 6. Therapies under Evaluation

Supportive care and prevention of bacterial superinfection are the mainstay of management in patients with SARS-CoV-2 infection. Many off-label and compassionate-use pharmacologic therapies are being used without substantial evidence from high quality clinical studies and many of these treatments are currently undergoing evaluation in clinical trials.

### 6.1. Antivirals

Antivirals aim at blocking viral replication and related cell damage. Even though antivirals seem to be the most obvious causative therapy, their efficacy might be limited to the initial phase of the illness and might not have any effect in the advanced stages, when inflammatory mechanisms seem to prevail. Antivirals that have been used or proposed in Covid-19 include lopinavir/ritonavir, remdesivir, ribavirin, and favipiravir.

So far, lopinavir/ritonavir has been the only antiviral agent undergoing a major randomized clinical trial. Lopinavir/ritonavir is an approved oral combination agent for the treatment of HIV. The association has been previously used in SARS and retrospective studies seemed to suggest a reduction in mortality and intubation rates in patients who received this treatment [142]. The first published randomized trial in Covid-19 was open-label and included 199 hospitalized adult patients with confirmed SARS-CoV-2 infection. No adjunctive benefit in the time to clinical improvement, mortality rates at 28 days and viral RNA load at various time point was observed in patients who received lopinavir/ritonavir (n = 99) compared to patients who were managed as per standard of care (n = 100) [143].

Remdesivir is a nucleotide analogue prodrug that inhibits viral RNA polymerases. In vitro testing of remdesivir has shown a potent activity against SARS-CoV-2 [144]. A small cohort of 53 patients hospitalized for severe Covid-19 were treated with remdesivir on compassionate-use bases [145]. Clinical improvement in oxygen-support status was observed in 36 of 53 patients (68%). The results of one randomized, double-blind, placebo-controlled, multicenter trial that included 237 patients with Covid-19 from China admitted to hospital with an interval from symptom onset to enrolment of 12 days or less and pneumonia, showed a small reduction in the time to clinical improvement with remdesivir but failed to find statistical significance [146]. Mortality was also similar among treated and non-treated patients. This study failed to enroll the predetermined number of patients. Preliminary analysis from another randomized, controlled trial involving 1063 patients in the United States have also been recently made available. Preliminary results indicate that patients who received remdesivir had a 31% shorter median time to recovery (11 days versus 15 days) compared with placebo (*p* < 0.001). Also, in the group receiving remdesivir a survival benefit was reported with a mortality rate of 8.0% versus 11.6% for the placebo group (*p* = 0.059). The trial closed to new enrollments on April 19 and more comprehensive results will soon be available (NCT04280705). While interesting, these data still suggest that the role of antiviral treatment may be limited, with a greater efficacy possibly to the early phases of infection, likely due to the rapid replication of the SARS-CoV2 virus.

Favipiravir is a nucleotide prodrug whose active compound inhibits viral RNA-dependent RNA polymerase. Favipiravir is approved for the treatment of influenza virus infections in Japan and China. The results of a small open-label non-randomized control study conducted in China in Covid-19 have been published recently [147]. In the study patients receiving favipiravir plus inhaled IFN (n = 35) were compared with an historical cohort of patients who had been treated with lopinavir/ritonavir during the prior weeks (n = 45). Patients in the favipiravir group had faster viral clearance (4 days vs. 11 days) and more frequent radiographic improvement (91% vs. 62%). Still under review, another Chinese open-label, randomized study showed that moderately ill patients (but not mildly nor severely ill patients) treated with favipiravir had higher clinical recovery rates at day 7 compared to patients treated with umifenovir, a membrane-fusion inhibitor active against influenza [148]. More trials are underway.

Ribavirin has been empirically included into various treatment protocols for Covid-19 even though there is little evidence for its efficacy [149]. Ribavirin is a nucleoside analogue that inhibits viral RNA-dependent RNA polymerase with in vitro activity against SARS-CoV only at high concentrations. Ribavirin has been used for the treatment of SARS and MERS, mostly in combination with IFNs. Of 30 trials evaluating ribavirin in patients with SARS, 26 were classified as inconclusive and 4 reported possible harm due to the occurrence of hemolytic anemia and liver toxicity in a high proportion of treated patients [150]. With these premises ribavirin likely has limited value for the treatment of Covid-19.

### 6.2. Chloroquine and Hydroxychloroquine

Chloroquine and hydroxychloroquine inhibit in vitro SARS-CoV-2 [151]. These agents appear to interfere with viral entry into cells as well as viral replication. In addition, they attenuate cytokine production and inhibit autophagy and lysosomal activity in host cells [151]. Given the long history of use in patients with malaria as well as in patients with rheumatologic conditions, and considered the lack of alternatives, the use of hydroxychloroquine, alone or in combination with azithromycin (an antibiotic added mainly for its anti-inflammatory effects possibly due to attenuation of IL6), for the treatment of Covid-19 has widely spread despite very limited efficacy data and emerging concerns of cardiotoxicity, especially for the association of the two drugs. The only trial published so far is a small single center open label study on 36 patients with Covid-19. The primary outcome in the trial was viral clearance from the nasopharynx, not a clinical outcome. At day 6, 70% of patients who received hydroxychloroquine (n = 20) achieved viral clearance compared to 12.5% of patients in the control group (n = 16) [152]. The authors also pointed out the potential synergistic effect of the concomitant use of azithromycin since all the patients receiving the combination achieved viral clearance (n = 6). Following the publication, attention was drawn toward several design and methodological flaws of the study and the scientific validity of the findings have been questioned [153]. Beside the small sample size and the fact that no clinical or safety outcomes were reported, several confounding factors were observed including the fact that six patients in the hydroxychloroquine group that met the inclusion criteria were removed from the analysis due to cessation of treatment as a consequence of worsening illness or medication-related adverse effects. Another, still unpublished small clinical trial randomized 62 patients to receive hydroxychloroquine or placebo and found a reduction of time to clinical recovery. This work, nevertheless, did not stratify patients for comorbidities and the clinical endpoints were not clearly defined [154].

Pending peer review, the results from several other works have emerged which question the safety and efficacy of this treatment. A retrospective analysis of data from 368 hospitalized patients with Covid-19 across the United States investigated the risk of death and the need for mechanical ventilation based on exposure to hydroxychloroquine alone or with azithromycin, placing a cautionary note. In the study, hydroxychloroquine, either with or without azithromycin, did not reduce the risk of mechanical ventilation. Moreover, an increased overall mortality was identified in patients treated with hydroxychloroquine alone [155]. Similarly, in a retrospective analysis of hospitalized patients evaluating probability of intensive care transfer or death, by means of an inverse probability of treatment weighting approach, did not find evidence of hydroxychloroquine efficacy [156].

On 21 April, the National Institute of Health expert panel that issued Covid-19 treatment guidelines recommended against the use, outside clinical trials, of the combination of hydroxychloroquine plus azithromycin, because of the potential for toxicities. Also, it was recommended to pay attention toward patients receiving chloroquine or hydroxychloroquine for adverse effects, especially prolonged QTc interval.

### 6.3. SARS-CoV2-Specific Monoclonal Antibodies

Human monoclonal antibodies specific for the SARS-CoV-2 virus might represent a possible passive serotherapy for selected patients. Several SARS-CoV monoclonal antibodies with in vitro neutralizing activity were generated from B lymphocytes of patients that recovered from the 2003 epidemic. A single human monoclonal antibody with cross-neutralizing activity against SARS-CoV and SARS-CoV-2 was recently reported [157], however no clinical experience or recruiting trials are available at the moment.

### 6.4. Immunomodulatory Agents

Significantly increased amounts of several proinflammatory cytokines driving the uncontrolled immune inflammatory response of Covid-19 have been measured in serum of patients who develop severe or fatal disease [158]. Which mediators have the most important role in immunopathology is still to be clarified. Several biological agents targeting inflammatory cytokines and cytokines receptors have been proposed as potential candidates.

Preliminary observations have been reported with the use of tocilizumab, a humanized monoclonal antibody targeting IL-6 receptor, approved for the treatment of cytokine-release syndrome following chimeric antigen receptor T-cell (CAR-T) therapy [159]. In small case series, the repeated administration of tocilizumab to patients with severe Covid-19 was associated with rapid cessation of fever, improvement of respiratory functions and thoracic imaging, as well as reduction of C-reactive protein [160,161]. A larger, prospective, case series on 100 patients admitted to hospital for Covid-19 pneumonia showed an improved or stable respiratory status in 77 patients within 10 days from treatment start [162]. Several randomized-controlled trials are currently under way to evaluate the role of tocilizumab and other monoclonal antibodies targeting IL-6 such as sarilumab and siltuximab. Preliminary data on compassionate use of siltuximab in 21 patients with pneumonia have shown improvement in 7, yet no conclusions can be inferred given the lack of control arm in the study [163]. It should also be considered that IL-6 has also significant anti-infective roles, therefore caution is advised in respect to the possibility that its inhibition can result in greater incidence of opportunistic infections in treated patients, especially when given together with corticosteroids.

Other anti-cytokine drugs seem to have a rationale for the treatment of Covid-19 associated inflammation. This is the case of anakinra, an interleukin (IL)-1 receptor antagonist used for various rheumatologic conditions, especially considering the aforementioned ability of SARS-CoV and MERS-CoV to induce NLRP3 inflammasome and IL-1beta. A recent report showed promising results using high doses of anakinra intravenously in 29 patients with severe disease [164], with rapid decrease of inflammatory markers, progressive amelioration of respiratory function and increased survival compared to a retrospective cohort, with a good safety profile. A second report on subcutaneous administration in nine patients [165] showed safety of the drug, with a slower response on inflammatory markers. Another experience of high intravenous doses also showed safety and efficacy in severely affected subjects [166]. Similarly, given the major role of inflammatory macrophages in Covid-19 immunopathogenesis, emapalumab, an anti-IFN-γ monoclonal antibody used for the treatment of hemophagocytic lymphohistiocytosis, has been proposed [167]. Both drugs are under evaluation in currently recruiting trials. In addition, anti-TNF-α monoclonal antibodies have been proposed as potential treatment to prevent progression to needing intensive care in Covid-19 [168]. Elements supporting a role of anti-TNF-α are the evidence of elevated TNF-α levels in patients with severe Covid-19 [158], and the biologic effects of anti-TNF-α therapy, which include a rapid decrease in both IL-1 and IL-6 levels [169], as well as the observation in preclinical model that anti-TNFs ameliorate the course of severe respiratory syncytial virus (RSV) and influenza in mice [170]. Notably, lack of TNF-α receptor as well as in vivo TNF-α neutralization resulted in protection against SARS-CoV morbidity and mortality in mice [65,171].

### 6.5. Convalescent Plasma, Hyperimmune Globulins, and Intravenous Immunoglobulins (IVIG)

Convalescent plasma has been used for various viral infections including H1N1 influenza, SARS, and MERS with some evidence of potential benefit [172,173]. Hyperimmune globulin products are used in adults and children to prevent or to treat viral infections such as cytomegalovirus, varicella zoster, and respiratory syncytial virus. Plasma from individuals who have recovered from Covid-19 containing antibodies to SARS-CoV-2 or specific antibody preparations derived from plasma may suppress viremia and modify the inflammatory response [174]. In two small case series of critically ill patients with Covid-19, administration of convalescent plasma containing neutralizing antibody was followed by clinical improvement in all patients without safety concerns [175,176]. No experience with hyperimmune globulin products in Covid-19 has been published so far. 

Small clinical experiences have been published with the use of donor pool intravenous immunoglobulin (IVIG) [7,177]. Their role is unclear, nevertheless IVIG may have both a partial inhibitory viral effect due to nonspecific cross-reaction, and a well-known immunomodulatory effect in several inflammatory conditions, including macrophage activation syndrome [178]. IVIG use may be most relevant in patients with specific organ involvement such as myocarditis and Guillan-Barré syndrome, which have also been reported in association with SARS CoV-2 infection [179].

### 6.6. Interferons and JAK-Inhibitors

Interferons are key molecules of innate antiviral response and might have a different role depending on the timing of administration, based on preclinical observation on a mouse model [65]. IFNs have been proposed as a potential treatment of Covid-19 for their in vitro and in vivo antiviral properties. So far, the use of inhalatory INF-α1b has been used in China but no clinical data are available regarding its efficacy. Observations in SARS and MERS on the combined use of IFN and ribavirin failed to show any improvement in mortality or viral clearance [150,180]. Given the previously discussed role of IFN in coronavirus diseases, treatment timing could represent the most determining factor. In mouse models, IFN-I administration within 1 day after infection protected mice from lethal MERS infection, while delayed treatment failed to effectively inhibit virus replication, and resulted in worsening of inflammatory changes in the lungs [49].

On the other hand, Janus Kinase (JAK) inhibitors have also been proposed to curb excessive IFN signaling in severe Covid-19. Baricitinib has attracted interest since, beside its immunomodulatory properties, it may reduce the ability of the virus to infect lung cells [181]. However, these drugs may also result in substantial depression of the immune response. A small, non-randomized study reported a cohort of 12 patients hospitalized for moderate Covid-19 pneumonia who were treated with baricitinib in addition to lopinavir/ritonavir. Compared to the previous 12 consecutive patients with moderate Covid-19 admitted before study start, who were treated with lopinavir/ritonavir and hydroxychloroquine, baricitinib-treated patients had a faster improvement of clinical conditions and respiratory parameters, did not require intensive care, and were discharged earlier. Notably, treatment was tolerated in all patients, with no serious adverse events or opportunistic infections [182]. Larger clinical trials are ongoing.

### 6.7. Corticosteroids

Corticosteroids have a rationale in Covid-19 for their potent anti-inflammatory effect and their potential role in suppressing cytokine-related lung injury. However, considerations have been made that beside suppressing lung inflammation, corticosteroids also inhibit immune responses and pathogen clearance. Observational studies in patients with SARS and MERS reported clinical efficacy but no clear association with improved survival, while demonstrating delayed viral clearance and high rates of complications [150,183,184,185,186]. In a recent meta-analysis evaluating the role of corticosteroids as adjunctive therapy in patients with severe pneumonia caused by influenza, corticosteroid therapy seemed to confer an increased risk of secondary bacterial infection and mortality [187]. It is noteworthy to report, however, that a recent COVID-19 series found that patients with ARDS treated with methylprednisolone exhibited lower mortality (46% vs. 62%, hazard ratio for death 0.38, *p* = 0.003) [188]. Based on these experiences, further studies assessing the role of corticosteroids in Covid-19 would be of extreme importance, and several clinical trials are ongoing. The U.S. National Institute of Health Covid-19 expert panel considered their use in severely ill patients reasonable on a case by case basis, taking into account factors as pre-existing medical conditions requiring chronic corticosteroid use, or hemodynamic status [189].

### 6.8. Other Therapeutic Strategies

The observation that viral entry into cells via ACE2 receptor requires priming by cellular proteases, mainly TMPRSS2, prompted in vitro trials with two clinically approved drugs for acute pancreatitis (nafamostat and camostat) which resulted in blockade of viral entry [16]. Another drug inhibiting TMPRSS2 is bromhexine, a widely used antitussive agent. Considering the role of TMPRSS2 in viral entry, these drugs could be suitable as prophylaxis in high-risk settings. All these three drugs are currently undergoing clinical trials.

The use of heparin for Covid-19-associated coagulopathy and its adjunctive potential anti-inflammatory role has been previously discussed [190].

## 7. Acquired Protection and Development of Vaccines

It is too early to be able to determine whether people can be re-infected by SARS-CoV-2 after recovery from Covid-19. Re-detection of viral RNA on nasopharyngeal swabs after two consecutive negative results was reported in asymptomatic patients during the convalescent phase [191]. However, since viral RNA shedding has been observed for days to weeks after resolution of symptoms [140,192], one possible explanation is that patients in whom SARS-CoV-2 RNA was re-detected had a false negative result due to an insufficient viral load of the specimen, or persistence of viral nucleic acids in the airways in the absence of the full, potentially infectious virus. 

Much of our understanding of the immune response to coronavirus in humans comes from observations in SARS-CoV and MERS-CoV. In longitudinal studies investigating the humoral immune response in patients who recovered from SARS and MERS, neutralizing antibodies against virus S protein were detectable for up to two years [193,194] in SARS-CoV, and up to three years in MERS-CoV [195]. Whether humoral immunity confers protection to reinfection remains to be established. A strong anti-spike antibody response appears to be protective in the susceptible host [196]. However, antibodies titer markedly declines already after one year and at 6 years only a minority of patients who recover from SARS show detectable IgG [197], suggesting that memory B-cells against SARS-CoV diminish over time. In recent cohort studies, antibodies against SARS-CoV-2 have been detected as early as the 4th day after symptom onset [198] and antibody levels do not appear to correlate with clinical severity. Notably, a significant proportion of patients (up to 30%) who recovered from Covid-19 develop low or absent neutralizing antibodies [9]. The reasons why coronavirus infections do not elicit robust and long-lasting antibody response are still unclear. 

According to some authors, T-cell response may be more important than the humoral response, both for recovering from primary infection and to prevent reinfections. Unlike antibodies, SARS-CoV-specific memory, CD8 T cells have been observed for up to 6 years post-infection [199,200,201,202,203,204]. Virus-specific CD8 T cells are required for pathogen clearance during acute infection. After that, memory CD8 T cells persist at anatomical front-line sites of specific microbial exposure [205] and, upon re-stimulation, are capable of proliferating and secreting effector cytokines (IFN, TNF, and IL-2) and cytotoxic molecules (granzyme B and perforin), and to recruiting other immune cells [206]. So far, most of our understanding is derived from experimental studies in animal models. In mouse models, enhancement of SARS-CoV specific CD8 T cells by immunization with viral peptide-pulsed DCs results in a robust T-cell response, earlier virus clearance, and increased mouse survival [207,208]. Recent observation in agammaglobulinemic patients of spontaneous recovery from SARS-CoV-2 infection supports the hypothesis that T-cell immunity might be more important than antibody response [71]. 

These observations cast doubts on whether the antibody response can be used as a correlate or surrogate of protection following wild infection. Also, it is likely that vaccines combining both humoral and cellular responses might be necessary for coronavirus prevention [196,199], with humoral immunity being relevant especially in the first phase of virus infection, reducing the initial viral load and controlling its spreading in respiratory organs, while cellular immunity being important in the control of the inflammatory phase of the disease. Furthermore, the efficacy of the above-mentioned convalescent plasma therapy suggests the potential role of humoral response elicited by a vaccine. More information on the protective role of both humoral and cellular compartments will probably be obtained from observation of the vaccine-induced immune response.

## 8. Conclusions

Despite huge amounts of data from thousands of research articles published each month, most knowledge on Covid-19 is derived from descriptive works. To date a possible therapeutic approach could involve an antiviral and a cell-entry inhibitor for the first phase and an immunomodulant such as a IL6 or IL1 blocker for the second phase if inflammation persists, with special treatment such as convalescent plasma, hyperimmune globulins or SARS-CoV-2-specific monoclonal antibodies reserved to selected patients. Even if a fairly defined picture is being formed that summarizes the importance of virologic, vascular, and inflammatory factors, much remains to be understood regarding the relationship of dependence between distinct pathological events during the course of infection. Consequently, the proposal of therapeutic interventions which may seem rational in the various pathological phases is still today largely based on hypotheses.

## Figures and Tables

**Table 1:** Critical mechanisms of potential relevance as targets of therapies.

Immunologic Mechanism Involved in Antiviral Response		Hypothesized Defects Associated with Severe Infection	Effect on Antiviral Response	Proposal of Possible Therapeutic Actions
Innate immunity	Early production of IFNs	Defects in IFN cascade.	Susceptibility to severe course of herpes, VZV and flu	Early Type-I IFN administration. Administration of effective antiviral medications
	Activity of natural killer cells	Defective cytotoxic functions	Primary hemophagocytic lymphohistiocytosis. Susceptibility to herpetic viruses.	Administration of third-party NK. Blockade of lymphohistiocytosis associated cytokine storm
	Late activation of neutrophils	Increased inflammasome signaling	Possible enhancement of inflammatory response to viruses	Anti-cytokine biologic agents
Adaptive immunity	Recruitment and expansion of virus-specific CD4 T cells	Combined immunodeficiencies with defects in the generation of T-cell and B-cell repertoire	Susceptibility to severe infections from various kind of viruses	Administration of third-party specific lymphocytes. Modulation of thymic function
	Killing of infected cells by virus-specific CD8 T cells	Defective cytotoxic functions and defects in generation of T-cell receptor	Defective response to most viruses	
	Production of antibodies	Agammaglobulinemia	Not strictly required to overcome the infection. Reduced memory response to new challenges from the same virus	Hyper-immune plasma from recovered subjects
		Production of ADE-capable antibodies	Increased spreading of infection, even to cells not expressing ACE2	Administration of high dose donor immunoglobulins
		Production of autoantibodies	Vasculitis	Vascular protection; anticoagulation; immunosuppression
